# Peptide–Oligonucleotide Conjugation: Chemistry and Therapeutic Applications

**DOI:** 10.3390/cimb46100655

**Published:** 2024-09-30

**Authors:** Anna L. Malinowska, Harley L. Huynh, Sritama Bose

**Affiliations:** Medical Research Council, Nucleic Acid Therapy Accelerator (UKRI), Research Complex at Harwell (RCaH), Rutherford Appleton Laboratory, Harwell OX11 0FA, UK

**Keywords:** peptide–oligonucleotide conjugates, conjugation chemistry, receptor-mediated oligonucleotide delivery, nucleic acid therapeutics, cellular uptake, cell-penetrating peptides, drug delivery

## Abstract

Oligonucleotides have been identified as powerful therapeutics for treating genetic disorders and diseases related to epigenetic factors such as metabolic and immunological dysfunctions. However, they face certain obstacles in terms of limited delivery to tissues and poor cellular uptake due to their large size and often highly charged nature. Peptide–oligonucleotide conjugation is an extensively utilized approach for addressing the challenges associated with oligonucleotide-based therapeutics by improving their delivery, cellular uptake and bioavailability, consequently enhancing their overall therapeutic efficiency. In this review, we present an overview of the conjugation of oligonucleotides to peptides, covering the different strategies associated with the synthesis of peptide–oligonucleotide conjugates (POC), the commonly used peptides employed to generate POCs, with the aim to develop oligonucleotides with favourable pharmacokinetic (PK) or pharmacodynamic (PD) properties for therapeutic applications. The advantages and drawbacks of the synthetic methods and applications of POCs are also described.

## 1. Introduction

One of the major factors limiting oligonucleotide-based therapeutics from reaching their full potential is the limited ability of these relatively large and often highly charged molecules to effectively cross cellular membranes, making it difficult for them to attain therapeutic concentrations at the site of action in the cytosol or nucleus [1,2]. Poor pharmacokinetic properties and cell permeability limit the application of this class of therapeutics. To realize the full potential of oligonucleotide therapeutics, the pharmacokinetic properties need to be improved, which can be achieved by conjugation with uptake-enhancing ligands that can modulate pharmacokinetic behaviour or target specific receptors. The chemical conjugation of oligonucleotides with transporter molecules is one of the most convenient approaches employed to improve the intracellular delivery and therapeutic potential of these agents [3]. Moreover, conjugation can also improve specificity by ensuring specific binding to a desired biological target. The commonly used molecules that are conjugated to oligonucleotides to increase cellular uptake are lipophilic compounds such as cholesterol, fatty acids, tocopherol and cell-penetrating peptides (CPPs). To achieve tissue-specific delivery, proteins for antigen-specific binding and small-molecule-based targeting moieties like carbohydrate *N*-acetyl galactosamine (GalNAc) have been used as oligonucleotide ligands. Despite recent advances, achieving efficient oligonucleotide delivery, especially to extra-hepatic tissues, remains a major challenge. In this review, we will be providing an overview of peptide–oligonucleotide conjugation as one of the key approaches to address the challenges associated with nucleic acid therapeutics. The progress in chemical approaches to peptide–oligonucleotide conjugation is regularly and thoroughly reviewed [4,5,6,7,8] and will be only briefly described here. Instead, we will focus on recent reports describing the conjugation of oligonucleotides to cell-penetrating and, especially, receptor-targeting peptides for the improved cell/tissue delivery of the conjugates and their potential therapeutic applications.

## 2. Discussion

### 2.1. CPP–Oligonucleotide Conjugates

Peptide–oligonucleotide conjugation has been extensively studied as a promising choice for oligonucleotide delivery, among which, cell-penetrating peptides (CPPs) have been widely used for oligonucleotide conjugation due to their ability to cross cellular membranes. CPPs are short peptides, usually containing fewer than 30 amino acids, that can penetrate biological membranes and allow the intracellular delivery of bioactive cargo molecules. Discovered in the 1990s, Tat peptide (derived from the transcription protein of HIV-1) [9,10] and penetratin (derived from the amphiphilic Drosophila Antennapedia homeodomain) [11] were the first reported examples of CPPs. Inspired by these molecules and focused mainly on positively charged sequences, thereafter, several CPPs with varied charges, polarities and structures have been designed [12]. Although their intracellular delivery mechanism has not been fully elucidated, CPP-based cellular uptake, mainly mediated by direct transduction or endocytosis, depends on the structural characteristics, type and concentration of CPPs or cargos and the cell types being treated. Based on their physicochemical properties, CPPs are broadly divided into three main classes: cationic, amphipathic and hydrophobic peptides.

Cationic CPPs predominantly consist of positively charged amino acid residues such as Arg, Lys or His. The key factors influencing the activity of cationic CPPs are the number and position of positively charged arginines present [13]. Various studies have been carried out to determine the optimal requirement of the amount of positively charged amino acid residues. Based on these studies, it was found that peptides rich in Lys, His or Orn residues are less efficiently absorbed by cells compared to arginine-rich peptides due to the higher pKa of guanidine groups of arginine and their ability to form bidentate hydrogen bonds with negatively charged phospholipids, acidic polysaccharides and proteins that are present in the cellular membrane. Moreover, the minimum amount of Arg residues required is not less than 6, but, to ensure effective cellular uptake, the optimal amount is 8–10 residues [14]. Higher values can have detrimental effects on the cells and reduce overall delivery efficiency [15]. Most of the cationic CPPs are of natural origin; however, synthetic CPPs have also been developed and include arginine homopolymers, peptides of the Pipseries developed by the Gait group [16]. Under normal physiological pH conditions, the positive charge of cationic CPPs shows excellent affinity with the cytoplasmic membrane. The cationic CPP attaches itself to the negatively charged cell membrane glycoprotein through an electrostatic interaction and then internalises into the cell through a mechanism independent of the receptor. Currently, most of the existing CPPs consist of multiple positively charged arginine residues, which are often a source of toxicity [17]. As an example, Sun et al. [18] showed that among the arginine-rich peptides, (RRRRRRRRRRR), a peptide composed purely of arginine residues, has the lowest LD_50_ value (16.5 mg/kg) and manifests the highest toxicity, whereas (ACSSSPSKHCG), a peptide without arginine residue, shows much lower toxicity and higher survival rates in mice. Moreover, the ability of CPPs to translocate through cell membranes can be accompanied by toxic effects resulting from membrane perturbation at higher peptide concentrations. Clinical applications of CPPs also depend on the improvement of endosomal escape efficiency. Among the potential mechanisms for endosomal escape, one probable explanation is based on positively charged CPPs, which are thought to bind to negatively charged components in the endosomal membrane. This leads to the formation of a membrane pore, which results in the leakage of CPPs. Another possible reason for escape is the formation of ionic pairs between negatively charged phospholipids and positively charged CPPs, which would partition across the endosomal membrane.

Amphipathic CPPs are the most abundant class of peptides among the CPPs and make up about 40% of all CPPs. Amphipathic peptides consist of polar and non-polar amino acid regions, and the non-polar region contains hydrophobic amino acid residues such as alanine, valine, leucine, isoleucine, etc. Although most of the amphipathic CPPs are chimeric or synthetic, they can also be derived from natural proteins such as pVEC or ARF (19–31) [19]. Amphipathic CPPs can again be subdivided into three subclasses: primary, secondary and proline-rich CPPs. Primary amphipathic CPPs are usually chimeric peptides obtained by covalently binding hydrophobic amino acid domains with a nuclear localisation signal (NLS) such as MPG peptides [20] and Pep-1 [21], which are both based on the SV40 NLS [22]. Secondary amphipathic CPPs usually have α-helical conformation, with hydrophilic and hydrophobic residues grouped on opposite sides of the helix. A few examples of amphipathic peptides are MAP [23], transportan [24] and CADY [25].

Hydrophobic CPPs are the smallest class of CPPs and they contain non-polar or less-charged amino acid residues. Their mechanisms of cellular penetration are not fully elucidated, but it is believed that it occurs due to their high affinity for the hydrophobic domains of cell membranes. A few examples of hydrophobic CPPs are C105Y [26] and Pep-7 [27].

The interactions between a CPP and its cargo can be broadly classified into two types—covalent and non-covalent. For example, CPPs can be conjugated to their oligonucleotide cargo by direct covalent conjugation through a linker, such as via cationic or amphipathic CPP conjugation to a neutral-charge oligonucleotide like phosphorodiamidate morpholino oligonucleotide (PMO). The chemical methods for the synthesis of peptide–oligonucleotide conjugates are discussed in detail later in this review. On the contrary, non-covalent conjugation occurs through electrostatic and hydrophobic interactions between the CPP and the oligonucleotide to form nanoparticulate complexes that can pass through the cell membrane efficiently via endocytosis [28]. The initial example using the non-covalent strategy for oligonucleotide delivery was the MPG peptide [20], after which, it was extended to several other CPPs such as Pep-1 or Tat.

Peptide–oligonucleotide conjugates can be prepared via post-synthetic coupling, that is, the separate assembly of peptides and oligonucleotides using their respective automated solid-phase syntheses, and then performing the conjugation in a solid or solution phase. The alternative method is a stepwise solid-phase assembly in succession on the same solid support, commonly known as in-line solid-phase synthesis. For the latter, usually, the peptide part is first assembled using *t*-butoxycarbonyl (Boc) amino acids with base-labile protecting groups, avoiding the use of strong acids in the presence of the oligonucleotide [29]. However, in some cases, fluorenylmethoxycarbonyl (Fmoc) amino acids with Boc protection [30] or 1-(4,4-dimethyl-2,6-dioxocyclohex-1-ylidene) ethyl (Dde) [31]-protecting groups have also been reported. The stepwise method holds advantages in terms of purification and higher yields. Yet, there are certain disadvantages of this method arising due to the non-compatibility of peptides and oligonucleotides with the synthetic methods. On the other hand, post-synthetic conjugations require multiple steps involving the separate solid-phase synthesis of both partner compounds followed by purification, performing the conjugation with these two fragments, and subsequent purification, resulting in lower yields of the final product. Despite the limitations, the post-synthetic method is favourable and more frequently used due to the availability of a wide range of conjugation chemistry techniques that are mentioned here.

**Scheme 1:**
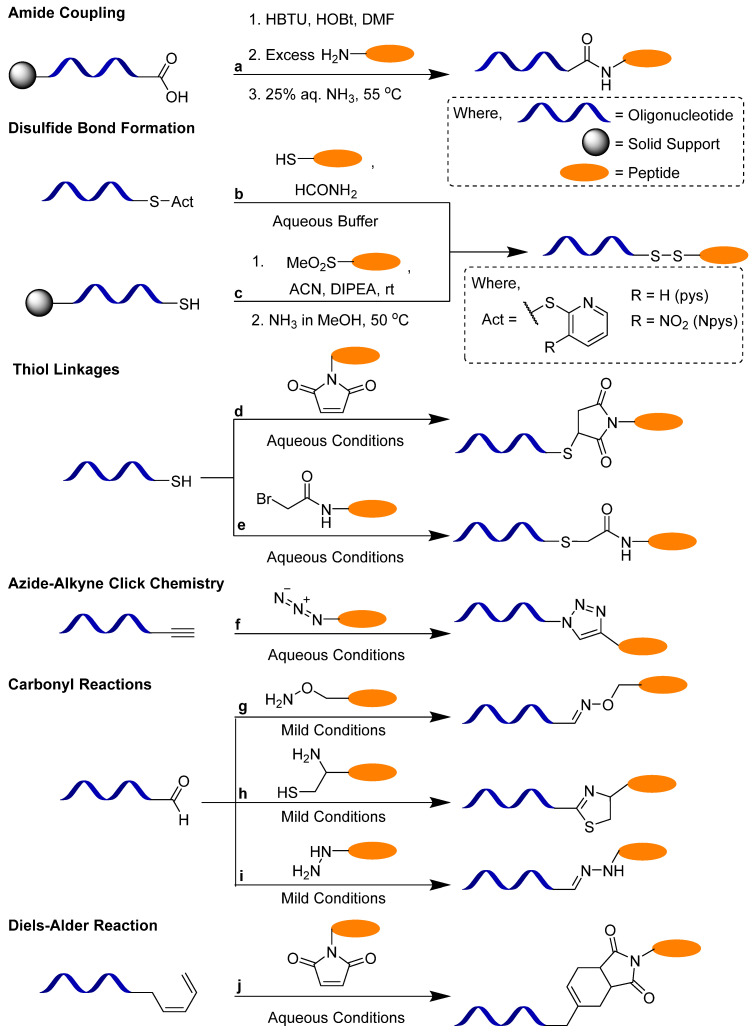
Post-synthetic methods for the synthesis of POCs. (**a**) Amide coupling reaction. (**b**) Disulfide activation reaction with thiol. (**c**) S-sulfonate-protected cysteine reaction with thiol. (**d**) Thiol–maleimido reaction. (**e**) Thiol–bromoacetamido reaction. (**f**) Azide––alkyne click reaction. (**g**) Oxime reaction. (**h**) Thiazolidine reaction. (**i**) Hydrazone reaction. (**j**) Diene–maleimido Diels–Alder reaction. Adapted from [6,8].

In the case of post-synthetic reactions, different methods have been used for peptide–oligonucleotide conjugations, among which, the most important are amide coupling [32], disulfide bond formation [33], thiol–maleimide click chemistry [34], oxime [35,36], hydrazone [37] or thiazole [36] bond formation and copper-catalysed azido–alkyne cycloaddition (CuAAC) click chemistry [38] (Scheme 1). Other novel post-synthetic methods include native chemical ligation [39], Diels–Alder reactions [40], nitrile–aminothiol conjugation [41] and metal-free thiol-ene click reaction with vinylpyrimidine linkers [42]. It is to be noted that the synthesis of siRNA–peptide conjugates is further challenging due to the labilityof small interfering RNAs (siRNAs) to basic conditions and the additional requirement for protection of the 2′-OH group. Although there are some reports in the literature regarding the preparation of siRNA–peptide conjugates by stepwise synthesis [43], in most cases, these conjugates are prepared by post-synthetic methods. Recently, Gothelf et al. [44] reported the synthesis of peptide–siRNA constructs by conjugation at internal phosphorus positions via sulfonyl phosphoramidate modifications. These modifications were beneficial as they could be directly incorporated into chemically modified oligonucleotides simply by changing the oxidation step during synthesis.

The conjugation of peptides to oligonucleotides through amide bond formation involves the reaction of an amino group with an activated carboxylic group. Amide conjugations can be performed using conventional amide coupling methods such as carbodiimide-mediated (HATU/HBTU/HOBt) [45] coupling, NHS ester activation or the acid chloride method. In this method, the oligonucleotide, while still attached to the solid support, can be detritylated to obtain the free carboxylic acid group, which can be activated by the coupling agents, and then the conjugation can be carried out with the N-terminus of the peptide. The reverse approach has also been successfully employed where the C-termini of amino acids and dipeptides have been conjugated with the N-terminus of the amino group of the oligonucleotide, using 1-ethyl-3-(3-dimethylaminopropyl)carbodiimide (EDC) as the coupling reagent [46]. A drawback of this method is the possibility of racemisation of the peptide.

The disulfide linkages can be synthesised by oxidation of two free thiol groups [47] or by modifying one of the thiols to form an activated disulfide [48,49]. Disulfide linkages are designed to be cleaved in the reductive environment in endosomes after successful cellular uptake [50]. Disulfide bridges can be specially used for peptide–oligonucleotide conjugates, for which a cysteine-containing amino acid can be used as a handle. Recently, another disulfide bond formation method for the synthesis of POCs was developed using S-sulfonate-protected cysteine of the peptide, both in a solution phase [51] and a solid phase [52]. S-sulfonates undergo thiolysis to afford disulfide-linked conjugates. Thiol linkers are also commercially available for attachment to oligonucleotides at the 5′-end during solid-phase synthesis and can also be attached to the amino linkers. Thiol functionalities can be used for preparing maleimide-type linkages, which are widely used for labelling proteins, proceed without a catalyst in aqueous buffers, and result in stable covalent linkages [53]. In the case of thioether bond formation, this occurs either via the Michael addition of thiols to maleimides or through the nucleophilic substitution of haloacetamides. The maleimido group can be introduced into the peptide or oligonucleotide using reagents containing activated esters such as β-maleimidopropionic acid [54]. For the synthesis of haloacetamides, either of the conjugating partners is modified with an aminohexyl group using halogenoacetic anhydride treatment [55].

Due to its specificity and efficiency, click chemistry is considered a useful method for the generation of bioconjugates. Azide–alkyne cycloadditions with or without copper catalysts can be carried out in aqueous buffers at room temperature [56]. Strömberg et al. developed a copper (I) bromide–dimethyl sulfide complex catalysed alkyne–azide cycloaddition method for the synthesis of phosphorothioate conjugates in high yields [38]. Copper-free click chemistry is the desired method as copper-based catalysts can be difficult to remove post-reaction and can lead to cytotoxicity. Linkers for introducing an alkyne- or dibenzo-cyclooctyne group during oligonucleotide synthesis are commercially available, and ligands can be easily derivatised for click reactions [57]. Azides and alkynes can be attached to terminal hydroxyl groups with phosphoramidite reagents or by coupling to amine linkers. Alkyne functionalities tethered to C5 of uridine can be employed for click reactions at the nucleobase.

Peptide–oligonucleotide conjugates linked by oxime, thiazolidine or hydrazone groups can be obtained by the reaction of carbonyl groups with an aminooxy group, 1,2-aminothiol and hydrazino, or hydrazido group, respectively. Usually, the reactions can be performed under mild conditions. Such conjugation methods have been reported extensively for synthesising POCs with oligonucleotides containing aldehyde or masked aldehyde groups at the 3′- or 5′-ends via thiazolidine [58], oxime [36] or hydrazone [59] formation. Recently, Virta et al. [60] reported an interesting approach for obtaining 2′-conjugates via *N*-methoxyoxazolidine formation. In this method, a peptide with an aldehyde group was conjugated to an oligonucleotide containing a 2′-*N*-methoxyamino and a free 3′-OH group. The stability of the conjugate was pH-dependent as the reaction was reversible under slightly acidic conditions, displaying properties of a cleavable linker that could release its cargo after entering cells via endocytosis.

The Diels–Alder reaction is a more novel approach that has been employed for the synthesis of POCs. Grandas et al. performed a Diels–Alder reaction between an acyclic diene at the 5′-end of the oligonucleotide and a maleimido peptide [61]. An inverse electron-demand Diels–Alder reaction was also applied for the synthesis of POCs, where 7-oxanorbornene was used as a dienophile and tetrazine was used as the diene [62]. The oligonucleotide or peptide fragments were derived from oxanorbornene-containing phosphoramidite or carboxylic acid, respectively.

The conjugation of CPPs to phosphorodiamidate morpholino oligonucleotides has shown promising results in terms of improving the pharmacokinetic profile of these molecules [63]. As PMOs have limited cell permeability, CPP conjugation has been used to improve cellular uptake and delivery, such as with the arginine-rich B-peptide (B-PMO), which demonstrated approximately 50% wild-type dystrophin levels following a single 25 mg/kg dose compared to the naked PMO that was administered weekly at 200 mg/kg for 12 weeks, achieving only 10% wild-type dystrophin levels. Peptide–PMO (PPMO) conjugates have been extensively studied for hereditary neuromuscular diseases such as Duchenne muscular dystrophy (DMD) [64]. Although unconjugated PMOs have been approved for DMD treatment, in some cases, low activity and poor delivery to muscles have been reported. For this purpose, PPMOs such as PGN-EDO51 (PepGen), SRP-5051 (Sarepta) and ENTR-601-44 (Entrada) are being explored for DMD treatment [65]. The Pip-6a PMO discovered by Wood et al. [66] is formed of a CPP containing a hydrophobic core flanked by arginine-rich domains consisting of β-alanine and aminohexanoyl spacers. In preclinical studies, it was demonstrated that compared to unconjugated PMO, the Pip-6a-conjugated PMO significantly enhanced antisense oligonucleotide (ASO) delivery into striated muscles of mice following systemic administration [67]. Amide conjugation methods have been used for connecting the C-terminus of the peptide with the 3′-terminal NH group of the PMO [68] using coupling reagents like HBTU/HOBt/DIEA. Other nucleic acid conjugates that have been investigated are peptide nucleic acids (PNAs) conjugated to peptides, especially CPPs. Peptide–PNA conjugates have demonstrated promising antibacterial activity [69] and they are usually synthesised by the stepwise method on the same solid support [70].

Although CPP–oligonucleotide conjugates have been extensively studied and, despite their widespread application, to date, no CPP-conjugated drugs have been approved by the FDA, and several clinical trials have been terminated, since these exhibited narrow clinical applications due to positive charge-induced non-target and systemic toxicity, endosomal sequestration, a lack of cell/tissue specificity, in vivo instability, rapid clearance and immunogenicity [32]. Another drawback of positively charged CPPs is that conjugation to negatively charged nucleic acids results in electrostatic interactions that can lead to self-aggregation and potential interference with oligonucleotide target binding. Moreover, peptide–oligonucleotide conjugates are challenging to synthesise due to the structure and solubility issues of both peptides and oligonucleotides, incompatible synthetic methods, and poor yields. There is no universal method for the synthesis of peptide–oligonucleotide conjugates and, in most cases, the choice of method and the conditions for synthesis are on a case-by-case basis. However, the increasing potential of oligonucleotide therapeutics and the need to enhance their targeted delivery by conjugation with peptides are paving the way for the development of more efficient methods for the preparation of peptide–oligonucleotide conjugates.

### 2.2. Receptor-Mediated Targeted Delivery of Peptide–Oligonucleotide Conjugates

**Figure 1:**
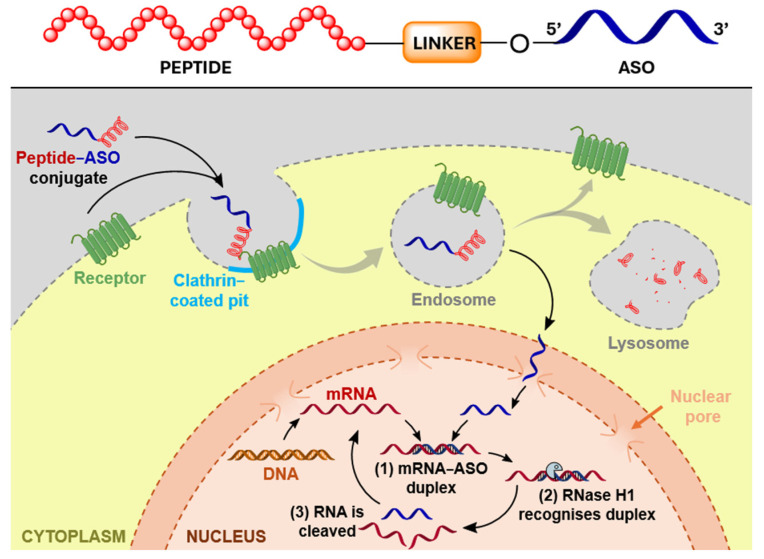
Mechanism of receptor-mediated targeted delivery of peptide–ASO conjugates. Adapted from [79].

There is an increasing interest in the targeted delivery of oligonucleotides via conjugation of the oligonucleotides to ligands binding specific receptors present at the plasma membrane of target cells [71]. This high-affinity interaction should ideally cause efficient internalisation, endocytosis, trafficking through endosomes (with ligand–receptor dissociation) and recycling of the receptor with the concomitant delivery and release of the cargo (oligonucleotide) (Figure 1) [71,72,73,74]. The flagship example of receptor-mediated delivery is conjugation of oligonucleotides to GalNAc, a carbohydrate moiety that binds to the asialoglycoprotein receptor (ASGPR) and facilitates the productive uptake of oligonucleotides into hepatocytes [75,76]. The conjugation of oligonucleotides to triantennary GalNAc leads to the successful clinical application of these molecules [77]. Following this example, other moieties, such as lipids, peptides, small molecules and antibodies, are being investigated as targeting ligands for extra-hepatic cell/tissue-specific delivery [3,8,78].

The main advantage of the application of peptides as targeting ligands is that, while being shorter, they can mimic the biological behaviour of full-length proteins, their chemical synthesis is more feasible and it is possible to introduce non-canonical and/or modified amino acids into the sequence. Selected examples of peptide-mediated selective tissue targeting are presented below.

The pancreas is a vital organ responsible for maintaining metabolic homeostasis in the human body, and the ability to deliver oligonucleotides to specific cell or tissue types within the pancreas would be extremely beneficial. In a proof-of-concept study, Ämmälä et al. explored the targeted delivery of ASOs to pancreatic β-cells through ligand-induced internalisation of the glucagon-like peptide-1 receptor (GLP1R) [72]. GLP1R belongs to the family of G protein-coupled receptors (GPCRs), which, as the largest family of human membrane proteins involved in maintaining various physiological processes, constitutes an important class of drug targets [80]. In the human pancreas, GLP1R is predominantly expressed in β-cells present within the pancreatic islets of Langerhans [81,82]. Glucagon-like peptide 1 (GLP1) is an endogenous intestinal peptide hormone responsible for the stimulation of insulin secretion and the inhibition of glucagon release [83], and GLP1 analogues (such as liraglutide and exenatide) have been extensively used in the treatment of type 2 diabetes (T2D) [81,82].

**Figure 2:**
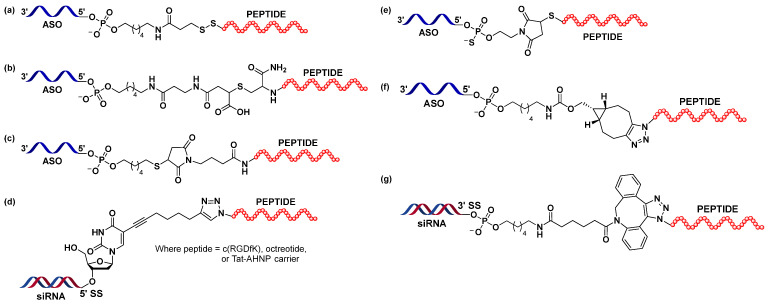
Various linker and spacer types used for conjugation of the peptides to oligonucleotides for receptor-mediated targeted delivery described in this review. Adapted from (**a**) Ämmälä et al. [72], (**b**) Knerr et al. [79], (**c**) Ming et al. [53], (**d**) Gandioso et al. [89], (**e**) Halloy et al. [90], (**f**) Nikan et al. [91] and (**g**) Broc et al. [92].

Finan et al. engineered a GLP1 analogue, a hybrid of two peptide sequences derived from human GLP1 (amino acids 1–29) and the C-terminal extension of exenatide (amino acids 30–39), which was used for the targeted delivery of estrogen to pancreatic islets [84,85]. Additionally, alanine at position 2 was substituted with 2-aminoisobutiric acid (Aib) to prevent proteolytic degradation by dipeptidyl peptidase IV (DPP-IV) [86], while glycine at position 16 was replaced by glutamic acid for improved potency [84,85]. Ämmälä et al. adopted the same peptide sequence by replacing C-terminal lysine (added as a handle for conjugation) with cysteine carboxyamide, which gave rise to eGLP1 peptides [72]. eGLP1 was attached at the 5′-end of the ASO gapmers via two types of cleavable linkers: the disulfide linker and the DNA phosphodiester T^m^CA [d(T^m^CA)] linker (Figure 2a) [87,88], employed to ensure the release of the free ASO inside cells, and separated by an aminohexyl spacer. eGLP1 was attached to the ASO gapmers targeting metastasis-associated lung adenocarcinoma transcript 1 (MALAT1) and forkhead box protein O1 (FOXO1). Experiments conducted both in vitro and in vivo showed that conjugation to the eGLP1 contributed to the improved productive uptake of conjugated oligonucleotides, as shown at the mRNA and protein levels.

In a follow-up study, Knerr et al. focused on exploring the structure–activity relationship (SAR) of ASO-GLP1R peptide agonist conjugates [79]. Originating from previously described eGLP-Malat-1 ASO [72], the authors explored the impact of features such as targeting peptide sequence/length or the nature of the linker/spacer on the activity of the conjugates in vitro and in vivo. This study underlined the importance of the optimisation of linker and spacer chemistries in construct design to obtain compounds that would be more potent in vivo. Among the different linker systems tested, the ring-opened maleimide analogue was identified as a lead structure (Figure 2b), as the conjugates carrying this linker showed the highest efficacy in vivo. Moreover, the results suggested that an additional d(T^m^CA) linker was not necessary, as the PO linker was sufficient for the conjugates to be active in mice. The study highlighted the complexity of conjugate design associated with discrepancies between the results obtained from experiments performed in vitro and in vivo and stressed the importance of the latter. The new maleimide acid-base linker design was also used to deliver ASOs against Islet Amyloid Polypeptide (IAPP) mRNA in mouse pancreatic β-cells and showed robust and sequence-specific target inhibition. IAPP (amylin) is co-expressed and co-secreted with insulin by β-cells [93]. In an insulin-resistant state, the expression of IAPP is increased, which can lead to the formation of toxic aggregates. These amyloid deposits of IAPP are usually present in the diabetic islet of Langerhans and have been associated with β-cell dysfunction and disease. Gurlo et al. used eGLP1-IAPP-ASO to suppress the expression of IAPP in mouse and human β-cells to verify its potential therapeutic application in T2D [94]. While the tested conjugate decreased the expression of the target mRNA in mouse cells, both in vitro and in vivo, it seemed to have no noticeable effect on target levels in human islets.

The BB_2_ receptor, also known as the gastrin-releasing peptide receptor (GRPR), is a member of the mammalian bombesin receptor family, which, in turn, belongs to the GPCRs superfamily [95]. BB_2_ is widely expressed in the gastrointestinal and central nervous systems, where it regulates various physiological processes. The overexpression of BB_2_ has been associated with numerous cancers, such as prostate, breast or small lung cancer [96,97,98]. Being an amphibian counterpart of mammalian gastrin-releasing peptide (GRP), the bombesin peptide is known to activate bombesin receptors. Conjugation to bombesin agonists and antagonists has been widely used for the selective delivery of chemotherapy agents or radioligands for cancer treatment and detection, respectively [98,99,100]. Ming et al. conjugated bombesin analogues to deliver splice-switching oligonucleotides (SSOs) to GRPR-positive PC3 prostate cancer cells to correct the aberrant splicing of the firefly luciferase reporter gene [53]. The bombesin peptide analogue (BBN; amino acids 6–14 of the original bombesin peptide sequence) was attached post-synthetically at the 5′-end of the oligonucleotide (2′-OMe phosphorothioate 20-mer) carrying a thiol linker via maleimide chemistry (Figure 2c). The study showed that treatment of the cells with conjugates resulted in a significant increase in luciferase gene expression when compared to the treatment with unconjugated oligonucleotides with the same sequence. BBN-SSO was primarily taken up by receptor-mediated endocytosis and was trafficked to deep endomembrane compartments. Contrary to lipofectamine-supported oligonucleotide delivery, the gymnotic delivery of the peptide conjugate resulted in a gradual increase in luciferase expression, peaking at 72 h, suggesting the engagement of different delivery mechanisms.

Gandioso et al. employed CuAAC click chemistry to attach three different targeting peptides to the siRNA for selective receptor-mediated delivery to cancer cells [89]. The peptides investigated were cyclic RGD (arginine–glycine–aspartic acid) containing cyclopentapeptide c(RGDfK), octreotide and cyclic anti-HER2/neu peptide (AHNP) attached to a Tat cell-penetrating peptide (Tat-AHNP).

RGD peptide, especially in a cyclic form, is known to be a high-affinity ligand for the α_ν_β_3_ integrin receptor [101,102], and the conjugation of peptides containing the RGD sequence for the receptor-mediated delivery of oligonucleotides was reported previously [74]. Integrins are a superfamily of heterodimeric transmembrane cell adhesion receptors consisting of various α and β subunits [102]. The cellular function of integrins is to coordinate adhesion and interaction with the extracellular matrix (ECM) with cytoskeletal re-arrangements and intracellular signalling via signal transmission across the membrane upon binding of the ligand [101,102]. The dysregulation of integrins is associated with the pathogenesis of many diseases with altered angiogenesis or inflammation [103]. α_ν_β_3_ was found to be expressed on various tumours and tumour-associated vessels and to be engaged in invasion and metastasis [101,104]. Octreotide is a clinically approved analogue of the endogenous somatostatin hormone, a peptide hormone responsible for various inhibitory activities including the inhibition of hormone secretion and the suppression of proliferation [105,106]. While somatostatin can activate five somatostatin receptor subtypes (SSTR1-SSTR5) [107], it has been shown that cyclic octapeptide octreotide selectively binds to the somatostatin subtype-2 (SSTR2) receptor (and, to a lesser extent, SSTR5), which is predominantly expressed in neuroendocrine tumours. Human epidermal growth factor receptor 2 (HER2), also known as erbB2, belongs to the family of receptor tyrosine kinases and is engaged in the activation of downstream signalling pathways related to cell proliferation, angiogenesis and survival [108,109]. HER2 is overexpressed in 25–30% of breast cancers, and drugs targeting this receptor (such as humanised recombinant monoclonal antibody Herceptin) have been developed for the treatment of the subset of HER2-positive breast cancers. AHNP is an exocyclic peptide that was designed to mimic the CDR3 loop of Herceptin [110,111]. As described in the previous section, TAT (Transactivating transcriptional activator) was the first CPP discovered; Tat-AHNP has been shown to efficiently penetrate HER2-positive breast cancer cells [112].

As stated above, all of these receptors are frequently over-expressed in tumours. To perform the conjugation, deoxyuridine phosphoramidite with octadiyne functionality at C5 (5oU) was introduced at the 5′-end of the sense strand of the siRNA (Figure 2d) targeting endogenous HER2 mRNA, while peptides were derivatised with an azide functionality [89]. An optimised CuAAC click reaction was performed post-synthetically and allowed for efficient conversion to the desired products for each of the peptides. The ability of the newly synthesised siRNA–peptide conjugates to enter the selected cancer cells and induce knockdown of the target expression was then investigated. SK-BR-3 human breast cancer cells were used to assess the biological activity of Tat-AHNP- and octreotide-siRNA conjugates, while the melanoma SK-MEL-28 cell line was employed to test the c(RGDfK)-siRNA construct. As shown by Western blot analysis, all conjugates were able to induce downregulation (although to a different extent and at high doses), which confirmed their productive uptake and activation of the RNAi machinery.

A bone marrow-homing heptapeptide [113] was tested, among other targeting moieties, for the delivery of SSO to bone marrow in order to modulate ferrochelatase splicing in a mouse model of erythropoietic protoporphyria [90]. Erythropoietic protoporphyria (EPP) is a rare genetic disorder caused (in the majority of patients) by a mutation in a gene encoding ferrochelatase (FECH), resulting in decreased levels of ferrochelatase enzymes involved in heme biosynthesis. Consequently, protoporphyrin is accumulated in the erythrocytes, plasma, skin and liver [114,115]. The retention of photoporphyrin in the skin leads to acute episodes of photosensitivity. Even a small increase in FECH synthesis could result in a therapeutic benefit, hence the idea of correcting the aberrant splicing with the use of SSO. However, this would require the delivery of oligonucleotides to bone marrow. Peptide was conjugated at the 5′-end of the phosphorothioate 2′-*O*-methoxyethyl (PS MOE) oligonucleotide via thiol–maleimide chemistry (Figure 2e) [90]. Although conjugation to the peptide increased the accumulation of the SSO in bone marrow, the splicing of the FECH transcript was not significantly improved, illustrating that increased delivery is not always followed by improved potency of the oligonucleotides.

The strain-promoted azide–alkyne cycloaddition (SPAAC) reaction [116] was applied to attach neurotensin peptides to ASO in order to investigate its influence on the productive cellular uptake and activity of the conjugates in vitro and in vivo (Figure 2f) [91]. Additionally, a thorough study of the structure–activity relationship of the conjugates was performed, taking into consideration features such as ASO length, ASO and peptide charge or linker type. The 13-amino-acid neuropeptide neurotensin (NT), found in the central nervous system (CNS) and gastrointestinal tract [117], was shown to be implicated in various biological processes including the modulation of dopaminergic transmission or hypothermic and analgesic responses [118]. NT binds with high affinity to two G-protein coupled receptors (NTS1 and NTS2) and the type 1 family receptor sortilin/NTS3 present in the plasma membrane of target cells [117,119]. As a receptor predominantly present in neurons of the central and peripheral nervous systems, sortilin plays an important role in regulating neuronal viability and function [118]. However, its engagement in the pathogenesis of CNS, vascular and metabolic diseases has been also reported [117,120]. 

The general synthetic strategy to obtain NT-oligonucleotide conjugates was as follows: phosphodiester (PO) MOE ASOs modified at the 5′-end with hexylamine was reacted with bicyclo[(6.1.0)]nonyne (BCN)-*N*-hydroxysuccinimide ester (NHS) [91]. The azide was introduced at the N-terminus of the neurotensin (KNT) [121]. To achieve this, N-terminal pyroglutamic acid residue was replaced with lysine. Finally, an SPAAC reaction between the modified peptide and oligonucleotide under mild conditions was performed (Figure 2f). It was shown that KNT-ASO was not internalised by the sortilin receptor expressing human embryonic kidney 293 (SORT1 HEK293) cells, possibly due to the undesired interaction between the positively charged amino acid residues in NT and the polyanionic backbone of the ASO. Conjugation of the NT to the PMOs with a neutral backbone resulted in increased uptake of the conjugates. Next, an SAR study was performed on the PO MOE ASOs to investigate the structural determinants of sortilin receptor-mediated uptake in cells. The key findings were that (1) the length of the ASO played a key role, as shortening the oligonucleotide improved uptake (with the shortest oligonucleotide showing the highest uptake); (2) the replacement of Arg8 and Arg9 (present in the binding site of NT) with lysine did not improve the activity of the conjugates, and Arg at positions 8 and 9 was crucial for NT interaction with the receptor; (3) long and flexible linkers did not augment the uptake; and (4) the insertion of a more rigid nine-amino-acid-long peptide linker at the N-terminus of NT (eNT) contributed to the improved internalisation of the conjugate. The latter eNT ligand design was then used for conjugation with the PS ASO gapmer to explore its effect on potency in the CNS in vivo. Oligonucleotides targeting the Malat1 RNA with 3-10-3 cEt BNA (2′,4′-constrained ethyl bicyclic nucleic acids) chemistry with a d(T^m^CA) linker at the 5′-end were used. The eNT-Malat1 conjugate demonstrated higher potency in the in vitro activity assay as well as in the spinal cord of the treated mice; no improvement was observed in the cortex, striatum or cerebellum. Lastly, the effect of neurotensin-mediated delivery on the potency of splice-modulating morpholino ASOs was evaluated. A previously reported morpholino ASO, known to correct survival motor neuron (SMN2) splicing, was used. Conjugation of the KNT to the morpholino ASO contributed to a modest increase in the potency of the oligonucleotide in mice brains.

Along similar lines, the angiotensin II peptide was attached to the ASO for improved delivery to extrahepatic tissues [122]. The peptide hormone Angiotensin II (Ang II) is a key bioactive molecule in the renin–angiotensin system (RAS) responsible for the regulation of blood pressure and plasma volume [123]. Angiotensin II acts through binding to its receptors, angiotensin II receptor type 1 (AGTR1) or angiotensin II receptor type 2 (AGTR2), with AGTR1 being the principal receptor for Ang II, expressed predominantly in the heart, adrenal gland and kidney [124,125]. Binding of Ang II to AGTR1 results in vasoconstriction, sodium and water retention and vasopressin release [125,126]. However, recent studies show that Ang II may also be involved in processes such as inflammation and ageing [123].

Also, in this case, SPAAC was employed to attach the Ang II peptide to PO MOE ASO [122], following an analogous synthetic strategy, as for neurotensin [91]. To make the Ang II peptide suitable for conjugation, azide–acetyllysine was added either at its N- or C-terminus. Additionally, a conjugate with a previously reported nine-amino-acid proline-rich linker at the N-terminus of Ang II (extended Ang II, eAng II) was prepared. This linker was introduced to diminish the charge interaction between the peptide and ASO and increase the rigidity. Contrary to conjugation via the C-terminus, conjugation through the N-terminus of the peptide resulted in the robust internalisation of the conjugates in the cell uptake study [(HEK293 cells stably expressed β-fluorogen-activating peptide (FAP)-tagged AGTR1)], with eAng II-ASO showing the best results. Next, Ang II/eAng II with an azido group at the N-terminus was attached to PS ASO [3-10-3 cEt BNA design with a d(T^m^CA) linker at the 5′-end] targeting Malat1 mRNA. Both conjugates showed improved potency in AGTR1-expressing FAP cells and mouse Purkinje-like cardiomyocytes. When administered to mice, a moderate increase in activity was observed for conjugates in the heart, adipose and adrenal gland, based on qPCR data. However, in situ hybridisation (ISH) analysis showed more pronounced activity in these tissues, especially in adrenal tissue, which may suggest a cell type-specific enhancement of ASO activity.

The low-density lipoprotein receptor (LDLR) represents an attractive cell surface target for siRNA delivery as it plays a key role in LDL–cholesterol plasma clearance [127]. Broc et al. [92] identified a library of peptide-based vectors that target the LDLR. The authors developed several VH4127 peptide–siRNA conjugates targeting the ubiquitously expressed superoxide dismutase 1 (SOD1) mRNA and explored the binding properties of these conjugates to LDLR and their efficiency in delivering siRNA into the cells. The peptide–siRNA conjugates were synthesised either by a copper-free click reaction (SPAAC) (Figure 2g) or by a direct amide coupling method using HATU. Although the LDLR-binding peptide was bulky and charged due to the presence of constrained cyclic octapeptides and arginine residues, it maintained its binding potential when conjugated to a siRNA and supported efficient uptake, resulting in appreciable gene knockdown both in vitro and in vivo.

Presented above are a few important examples of peptide–oligonucleotide conjugation for the receptor-mediated targeted delivery of oligonucleotides, highlighting the importance of this class of conjugates in the search for new, improved, oligonucleotide-based therapeutics.

## 3. Conclusions

The field of oligonucleotide therapeutics has witnessed great progress over the last few years, as reflected by a steady increase in the number of clinically approved therapeutics of this class. Although the use of GalNAc conjugation is a significant breakthrough, specific delivery to extra-hepatic cells/tissues, as well as productive uptake of the oligonucleotides, remain challenging. Therefore, the search for ‘new GalNAc’ is ongoing, and many researchers are working on the development of novel conjugates. Imetelstat (brand name Rytelo) is the first lipid–oligonucleotide conjugate and was recently approved for clinical use [128,129]. This first-in-class telomerase inhibitor found application in the treatment of myeloid hematologic malignancies. Although peptide–oligonucleotide conjugates have reached clinical trials, none of the representatives of this class has been clinically approved so far, but that has the potential to change with further research in this field.
